# Comparative Methylome Analysis of *Campylobacter jejuni* Strain YH002 Reveals a Putative Novel Motif and Diverse Epigenetic Regulations of Virulence Genes

**DOI:** 10.3389/fmicb.2020.610395

**Published:** 2020-12-23

**Authors:** Sandeep Ghatak, Cheryl M. Armstrong, Sue Reed, Yiping He

**Affiliations:** ^1^Division of Animal Health, ICAR Research Complex for NEH Region, Umiam, India; ^2^Molecular Characterization of Foodborne Pathogens Research Unit, Eastern Regional Research Center, Agricultural Research Service, United States Department of Agriculture, Wyndmoor, PA, United States

**Keywords:** *Campylobacter jejuni*, methylome, methylation, restriction-modification system, virulence gene

## Abstract

*Campylobacter jejuni* is a major cause of foodborne gastroenteritis worldwide inflicting palpable socioeconomic costs. The ability of this pathogen to successfully infect its hosts is determined not only by the presence of specific virulence genes but also by the pathogen’s capacity to appropriately regulate those virulence genes. Therefore, DNA methylation can play a critical role in both aspects of this process because it serves as both a means to protect the integrity of the cellular DNA from invasion and as a mechanism to control transcriptional regulation within the cell. In the present study we report the comparative methylome data of *C. jejuni* YH002, a multidrug resistant strain isolated from retail beef liver. Investigation into the methylome identified a putative novel motif (CGCG**A**) of a type II restriction-modification (RM) system. Comparison of methylomes of the strain to well-studied *C. jejuni* strains highlighted non-uniform methylation patterns among the strains though the existence of the typical type I and type IV RM systems were also observed. Additional investigations into the existence of DNA methylation sites within gene promoters, which may ultimately result in altered levels of transcription, revealed several virulence genes putatively regulated using this mode of action. Of those identified, a flagella gene (*flhB*), a RNA polymerase sigma factor (*rpoN*), a capsular polysaccharide export protein (*kpsD*), and a multidrug efflux pump were highly notable.

## Introduction

Campylobacteriosis is one of the most frequently acquired bacterial infections associated with the consumption of contaminated foods. Symptoms of campylobacteriosis include diarrhea, abdominal pain, vomiting, cramps, and fever. Although the infection is often self-limiting, its prevalence is a cause of concern for the World Health Organization (World Health Organization, 2020). Most cases of campylobacteriosis are caused by *Campylobacter jejuni*, a bacterium that exists as a commensal organism in the gastrointestinal tract of many domestic animal populations that are utilized as food sources such as poultry, cattle, pigs, and sheep (Facciola et al., 2017). Factors that influence the virulence of *Campylobacter* in humans are currently under investigation in an effort to devise better methods to control and/or prevent its spread.

DNA sequencing has become a driver for many scientific advancements over the past few decades. Sequence-based methods are now commonly used to identify bacterial virulence factors and as bioinformatic approaches advance, the frequency of utilizing comparative genomic analyses to identify genes involved with virulence continues to rise. Such analyses have already led to the identification of the pVir plasmid in *Campylobacter jejuni* (Bacon et al., 2002). With the rapid progress in whole genome sequencing (WGS) technology over last decade, the study of bacterial foodborne pathogens has gained new dimensions, which has provided further insight into the biology of these organisms (Llarena et al., 2017). In addition to generating genome sequences, Single Molecule Real Time (SMRT) technology offers a tool for studying epigenetic mechanisms, including DNA methylation. In bacteria, DNA methylation is a base modification system that is catalyzed by methyltransferases and results in the formation of the following: C^5^-Methyl-cytosine (m5C), N^6^-methyl-adenine (m6A), and N^4^-methyl-cytosine (m4C) (Sanchez-Romero et al., 2015). These modified bases enable the cell to identify and eliminate foreign DNA via the bacterial restriction-modification (RM) system, which has been shown to cleave double stranded DNA fragments. The resulting fragments are then further degraded by other endonucleases within the cell, essentially destroying any foreign DNA that may have been introduced that threatens the integrity of the cellular DNA. RM systems are common amongst bacteria with all four classes of RM systems (types I–IV) having been identified in *C. jejuni*^1^.

Methylation sites that are embedded within promoter regions or other regulatory sites may also serve as a mechanism by which genes, including virulence genes, can be regulated. For example, the presence of two DNA methylase targets within the regulatory region of the well-studied pyelonephritis-associated pilus (*pap*) operon has been shown to establish fimbrial phase variation in *Escherichia coli* populations by modifying the binding sites of a transcriptional regulatory factor involved in the process (van der Woude et al., 1996). It has been suggested by Mou et al. (2015) that DNA methylation may also be involved in the pathogenicity of *C. jejuni*, leading to some of the variations in disease presentation that can be seen amongst strains sharing a high degree of genomic synteny and sequence homology. This idea is further supported by gene products like that of cj1461, a N^6^-adenine-specific DNA methyltransferase that has been shown to play a role in factors related to pathogenicity including motility, adherence, and invasion in *C. jejuni* (Kim et al., 2008).

Overall, the study of methylomes is still in its infancy, with only a few reports available for *C. jejuni* (Murray et al., 2012; Mou et al., 2015; O’Loughlin et al., 2015) and none for a *C. jejuni* food isolate. In this context, we undertook the methylation analysis of the *C. jejuni* YH002, a strain isolated from beef liver, and compared/contrasted it with two well-studied *C. jejuni* strains to provide a deeper understanding into the biology of this important foodborne pathogen.

## Materials and Methods

### Detection and Analysis of Methylated Bases in *C. jejuni* YH002

The strain *C. jejuni* YH002 was isolated from retail beef liver using a passive filtration method (Speegle et al., 2009; Jokinen et al., 2012). The identity of the strain was confirmed by real-time PCR targeting the *hipO* gene (He et al., 2010). For genomic DNA preparation, the strain was grown in Brucella broth (Difco, Becton Dickinson, NJ) for approximately 40 h under microaerophilic conditions (5% O_2_, 10% CO_2_, and 85% N_2_) at 42°C and pelleted by centrifugation. High quality genomic DNA for SMRT sequencing was extracted and purified using the Qiagen Genomic-tip 100/G kit (Qiagen, Valencia, CA). Library preparation and SMRT sequencing was provided by the University of Delaware Sequencing and Genotyping Center (Newark, DE). The complete genome sequence and annotation are available in GenBank of NCBI with the accession No. CP020776 for the 1,774,584 bp chromosome and No. CP020775 for the 45,904 bp plasmid (pCJP002) (Ghatak et al., 2020).

Detection of methylated bases in the genome of *C. jejuni* YH002 was performed by using SMRT Analysis Suite 2.3.0^2^ with default parameters. Additional analyses were undertaken with various modified QV settings using the MotifMaker tool^3^.

The methylation locations in the *C. jejuni* YH002 genome were obtained by searching the 41 nucleotide (nt) context sequence of each motif in the raw PacBio data against the complete genome sequence. The average methylation frequency for each motif in the genome was calculated by dividing the number of occurrences of a particular methylation motif in either the chromosome or the plasmid by the total number of kilobase pairs in the chromosome (1,774.6 Kb) or the plasmid (45.9 Kb), respectively. All coding sequences (CDS) and gene products were predicted by RAST annotation^4^. The methylation frequency of the RAATTY motif in each CDS, rRNA, and tRNA was determined using a custom function added in Macros of Excel.

### Comparative Analysis Amongst *C. jejuni* Strains

For the comparative assessment of the methylomes, published motif data of *C. jejuni* 81–176 and *C. jejuni* NCTC 11168 were downloaded from REBASE database (Roberts et al., 2015). Genomes of *C. jejuni* 81–176 and NCTC 11168 were searched for motifs with a *P*-value cut off < 0.001 as described previously (Grant et al., 2011). Methylated and un-methylated CDS were then determined using RAST annotation and methylation data (Quinlan and Hall, 2010).

### Identification of Methylated Promoters

The locations of the transcriptional start sites (TSSs) in the *C. jejuni* YH002 chromosome were identified by sequence mapping the 50 nt region upstream of the TSSs in *C. jejuni* NCTC 11168, which were previously determined for *C. jejuni* NCTC 11168 using RNA-Seq data obtained from biological replicates during mid-log growth (Dugar et al., 2013). The *C. jejuni* YH002 chromosome is known to share high sequence similarity with *C. jejuni* NCTC 11168 (Ghatak et al., 2020). Similarly, the locations of the TSSs in the *C. jejuni* YH002 plasmid were identified by mapping the 50 nt sequences upstream of the TSSs in pTet in *C. jejuni* 81-176 since these two plasmids have 99% sequence similarity. In the present analysis, only those TSS deemed as either a primary or secondary TSS by Dugar et al. (2013) were utilized during the mapping process. All matched promoter sequences in *C. jejuni* YH002 were then checked for overlap with the methylated bases within the genome, ultimately deducing the promoter regions that were methylated (Quinlan and Hall, 2010). Plots of methylated and un-methylated CDS regions were generated using Circos (Krzywinski et al., 2009).

## Results

### Methylation Detection, Motifs, and Distribution Patterns

Detection of base modifications during SMRT sequencing of the *C. jejuni* YH002 genome identified putative methylation motifs (Table 1). Because generating motifs using the default settings in the modification analysis of the SMRT sequencing platform might not be ideal in all cases for motif identification (Methylome-Analysis-Technical-Note)^5^, we undertook a comparative analysis with various modification QV (modQV) cut-off values ranging from 30 to 200. Though the results (Table 1) indicated the presence of m6A and m4C types of modifications in the *C. jejuni* YH002 genome, low occurrences of m4C type modification, inconsistent detections, and their bizarre patterns were likely indications of spurious detection by the software (Motif maker) and therefore, we did not consider them as valid motifs in our results. Mean coverage of motif detection varied from 225X to 299X indicating satisfactory analysis. Overall, the results yielded 8–12 motifs depending on the modQV cut-off values. Among these, 7 motifs (Table 1) appeared consistently in all the analyses. Of the seven motifs, four motifs formed two pairs of bipartite motifs (herby named **A**CNNNNNCTC/G**A**GNNNNNGT and TA**A**YNNNNNTGC/GC**A**NNNNNRTTA), and another motif (RA**A**TTY) was palindromic. Search of REBASE database for similar motifs in *Campylobacter* genomes confirmed our results.

**TABLE 1:** Motifs identified in the genome of *C. jejuni* YH002 with various Modification QV (modQV) parameters^a^.

modQV cutoff	Motif string	Type^b^	Fraction (%)	Occurrence in genome	Partner motif	Mean IPD ratio^c^	Objective score
**30**	RA**A**TTY	m6A	99.0	30,196	RAATTY	5.839	11290815.0
	CCYG**A**	m6A	99.3	2,085	−	5.604	752000.4
	GKA**A**YG	m6A	99.1	1,555	−	5.950	613870.3
	**A**CNNNNNCTC	m6A	99.5	1,226	GAGNNNNNGT	5.449	446525.9
	G**A**GNNNNNGT	m6A	99.4	1,226	ACNNNNNCTC	5.992	439943.8
	TA**A**YNNNNNTGC	m6A	99.7	618	GCANNNNNRTTA	5.407	225513.5
	GC**A**NNNNNRTTA	m6A	99.0	618	TAAYNNNNNTGC	5.725	216201.3
	HNNNNN**A**RGTAAYG	m6A	57.9	164	−	3.443	6120.3
	GNNAG**A**GWANNNGND	m6A	43.3	104	−	3.330	515.1
	GC**G**GNAATNNNGNG	mod_base	100.0	6	−	2.462	363.0
	DGNNNN**A**CCTVAY	m6A	19.2	99	−	2.413	325.6
**40**	RA**A**TTY	m6A	98.9	30,196	RAATTY	5.840	11278810.0
	CCYG**A**	m6A	99.0	2,085	−	5.603	750188.0
	GKA**A**YG	m6A	98.8	1,555	−	5.957	612288.7
	**A**CNNNNNCTC	m6A	99.5	1,226	GAGNNNNNGT	5.449	446525.9
	G**A**GNNNNNGT	m6A	99.3	1,226	ACNNNNNCTC	5.996	439218.7
	TA**A**YNNNNNTGC	m6A	99.5	618	GCANNNNNRTTA	5.405	225145.7
	GC**A**NNNNNRTTA	m6A	98.9	618	TAAYNNNNNTGC	5.727	215850.6
	CA**G**NTAANCANNNNNNA	mod_base	77.8	9	−	1.914	366.1
**50**	RA**A**TTY	m6A	98.8	30,196	RAATTY	5.841	11266493.0
	CCYG**A**	m6A	99.0	2,085	−	5.603	750188.0
	GKA**A**YG	m6A	98.6	1,555	−	5.958	611083.9
	**A**CNNNNNCTC	m6A	99.3	1,226	GAGNNNNNGT	5.451	445776.1
	G**A**GNNNNNGT	m6A	99.2	1,226	ACNNNNNCTC	6.000	438851.8
	TA**A**YNNNNNTGC	m6A	99.0	618	GCANNNNNRTTA	5.403	224003.4
	GC**A**NNNNNRTTA	m6A	98.7	618	TAAYNNNNNTGC	5.726	215486.2
	G**A**GADNNNGMTR	m6A	52.7	186	−	2.468	5510.5
	DNNBN**A**CCTGAY	m6A	43.3	90	−	2.448	1770.6
	HNNNNACGCG**A**NANNNA	m6A	92.9	14	−	2.722	1673.6
**70**	RA**A**TTY	m6A	98.6	30,196	RAATTY	5.842	11236039.0
	CCYG**A**	m6A	98.6	2,085	−	5.605	746719.4
	GKA**A**YG	m6A	98.0	1,555	−	5.966	606910.3
	**A**CNNNNNCTC	m6A	98.9	1,226	GAGNNNNNGT	5.455	443441.6
	G**A**GNNNNNGT	m6A	98.8	1,226	ACNNNNNCTC	6.007	436927.9
	TA**A**YNNNNNTGC	m6A	98.7	618	GCANNNNNRTTA	5.405	223224.5
	GC**A**NNNNNRTTA	m6A	98.2	618	TAAYNNNNNTGC	5.734	214349.2
	WNNNN**C**RSGAATNT	m4C	36.2	149	−	3.571	4449.8
	G**A**GWDNNNGMTR	m6A	30.5	325	−	2.611	4048.9
**100**	RA**A**TTY	m6A	97.8	30,196	RAATTY	5.848	11142688.0
	CCYG**A**	m6A	97.4	2,085	−	5.608	736426.2
	GKA**A**YG	m6A	97.0	1,555	−	5.969	599839.9
	**A**CNNNNNCTC	m6A	97.7	1,226	GAGNNNNNGT	5.457	437677.1
	G**A**GNNNNNGT	m6A	97.5	1,226	ACNNNNNCTC	6.012	430320.7
	TA**A**YNNNNNTGC	m6A	97.7	618	GCANNNNNRTTA	5.409	220690.8
	GC**A**NNNNNRTTA	m6A	96.4	618	TAAYNNNNNTGC	5.753	209890.8
	**A**RGTAATGNNNNND	m6A	48.0	150	−	3.691	5429.8
	WNNNN**C**RSGAATNT	m4C	33.6	149	−	3.646	4023.6
	HHNNNNNACGCG**A**NNNB	m6A	43.8	80	−	3.375	2962.1
	**C**CCTGANNNRNANT	m4C	88.9	9	−	3.508	1441.8
**140**	RA**A**TTY	m6A	96.7	30,196	RAATTY	5.852	10980916.0
	CCYG**A**	m6A	95.7	2,085	−	5.620	720469.8
	GKA**A**YG	m6A	95.3	1,555	−	5.977	587514.6
	**A**CNNNNNCTC	m6A	96.4	1,226	GAGNNNNNGT	5.456	430640.8
	G**A**GNNNNNGT	m6A	95.9	1,226	ACNNNNNCTC	6.013	421956.2
	TA**A**YNNNNNTGC	m6A	95.3	618	GCANNNNNRTTA	5.408	214009.7
	GC**A**NNNNNRTTA	m6A	94.7	618	TAAYNNNNNTGC	5.757	205157.1
	DNNNN**C**RSGAATNT	m4C	28.3	159	−	3.809	3330.1
	HNNNNN**A**RGTAATG	m6A	29.3	150	−	4.008	2475.4
	CGCG**A**STNNANNNNND	m6A	100.0	8	−	4.424	2365.0
	DNND**C**CCTGAY	m4C	25.9	85	−	3.430	1375.3
	ACGCG**A**NNNNNNTNANW	m6A	100.0	6	−	3.118	1085.0
**170**	RA**A**TTY	m6A	96.0	30,196	RAATTY	5.855	10886096.0
	CCYG**A**	m6A	94.4	2,085	−	5.630	708218.3
	GKA**A**YG	m6A	94.1	1,555	−	5.979	578480.0
	**A**CNNNNNCTC	m6A	95.8	1,226	GAGNNNNNGT	5.456	426831.5
	G**A**GNNNNNGT	m6A	94.8	1,226	ACNNNNNCTC	6.036	415825.4
	TA**A**YNNNNNTGC	m6A	94.3	618	GCANNNNNRTTA	5.409	211109.1
	GC**A**NNNNNRTTA	m6A	92.9	618	TAAYNNNNNTGC	5.773	200120.3
	DNNNN**C**RSGAATNT	m4C	23.9	159	−	3.997	2560.3
	CGCG**A**STNNANNNNND	m6A	100.0	8	−	4.424	2365.0
**200**	RA**A**TTY	m6A	95.3	30,196	RAATTY	5.863	10775063.0
	CCYG**A**	m6A	92.9	2,085	−	5.646	691808.3
	GKA**A**YG	m6A	93.2	1,555	−	5.983	571029.3
	**A**CNNNNNCTC	m6A	95.5	1,226	GAGNNNNNGT	5.457	425312.0
	G**A**GNNNNNGT	m6A	93.9	1,226	ACNNNNNCTC	6.049	410360.9
	TA**A**YNNNNNTGC	m6A	92.2	618	GCANNNNNRTTA	5.424	204586.9
	GC**A**NNNNNRTTA	m6A	90.8	618	TAAYNNNNNTGC	5.784	193836.7
	DNNNN**C**RSGAATNT	m4C	28.2	103	−	4.008	2328.9
	CGCG**A**GTWNA	m6A	100.0	5	−	4.488	1530.0

**^*a*^Modified bases are boldfaced. ^*b*^mod_base: undetermined modification. ^*c*^IPD: Inter-pulse duration.**

Manual analysis of the remaining identified motifs (those not included in the consistently detected motifs described above) indicated that most were either a part of or an extension of those previously described motifs. However, a closer analysis revealed that among the set of motif strings which were not part of the motifs detected in all modQV runs was the putative hidden motif, CGCG**A**. We believe this to be a novel motif for *Campylobacter* species as our search in the REBASE database did not yield a match. However, an exact match was noticed for *Streptococcus mutans* (GenBank Accession CP044495), which was isolated from the oral cavity of a human.

**FIGURE 1:**
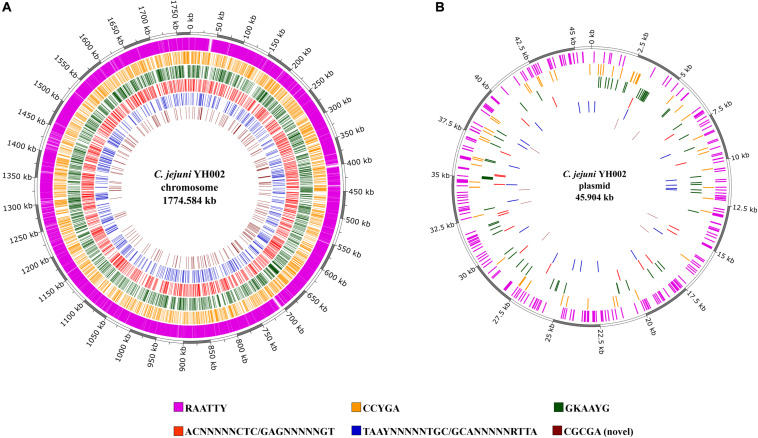
Methylation motifs within *C. jejuni* YH002. The locations of the methylated areas within both the **(A)** chromosome (GenBank accession No. CP020776) and **(B)** plasmid (GenBank accession No. CP020775) of *C. jejuni* YH002 are highlighted by their individual methylation motifs.

**FIGURE 2:**
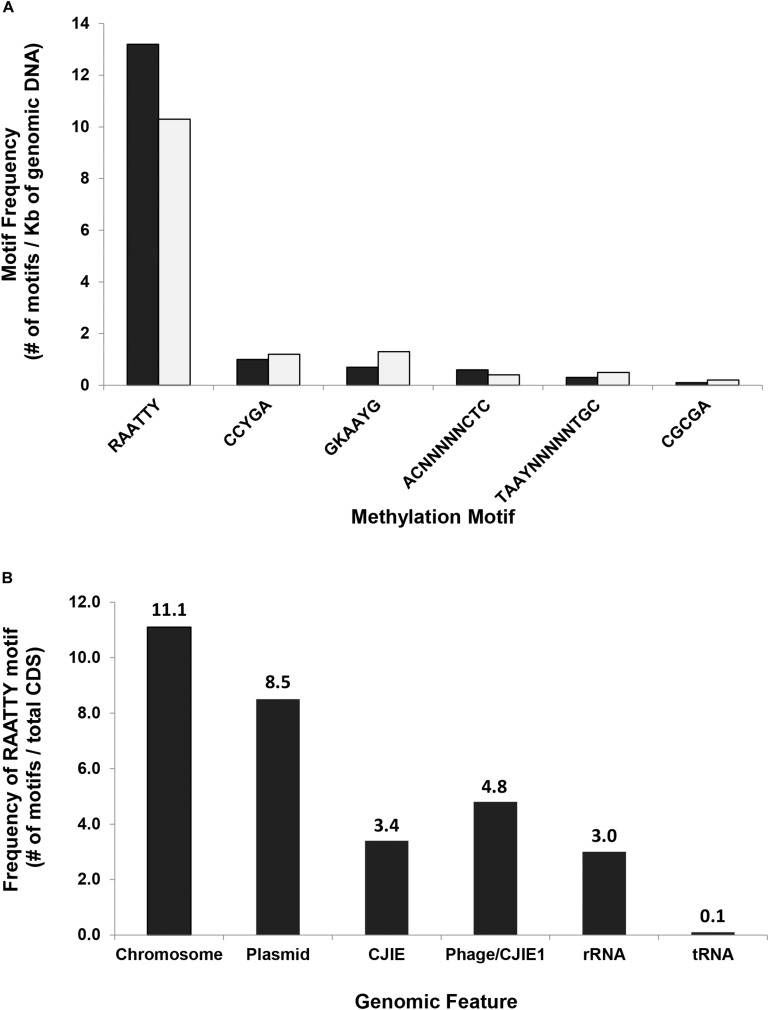
Methylation motif frequency in *C. jejuni* strain YH002. **(A)** The frequencies of six different methylation motifs in either the chromosome (black) or the plasmid (gray) were calculated using the number of a particular methylation motif divided by the total size of the chromosome (1,774.6 Kb) or the plasmid (45.9 Kb), respectively. **(B)** In-depth analysis of the frequency of the RA**A**TTY motif within different genomic features was determined using the number of RA**A**TTY motifs within a genomic feature divided by the total number of CDS in that genomic feature. Chromosome, *C. jejuni* YH002 chromosome; Plasmid, pCJP002; Phage/CJIE1, located between bp 1,409,078–1,444,627 in *C. jejuni* YH002;, and CJIE, Integrated mobile element located between bp 1,223,758–1,309,551 in *C. jejuni* YH002.

The consistently observed motifs were almost always (90.8–99.5%) methylated in the genome of *C. jejuni* YH002, with modification of the motif RA**A**TTY being the predominant motif in both the chromosome and the pCJP002 plasmid (Figure 1). This is in agreement with a similar analysis performed on a recently emergent *C. jejuni* sheep abortion clone known as *C. jejuni* IA3902 (Mou et al., 2015). A comparison of the frequencies associated with the different methylation motifs in *C. jejuni* YH002 demonstrated that both the RA**A**TTY and the **A**CNNNNNCTC/G**A**GNNNNNGT motifs showed a slightly higher frequency in the chromosome compared to the plasmid (Figure 2A). However, the frequency of the CCYG**A**, GKA**A**YG, TA**A**YNNNNNTGC/GC**A**NNNNNRTTA, and the CGCG**A** motifs were higher in the plasmid by comparison. Although the distribution of the RA**A**TTY motif appeared relatively uniform across both the chromosome and the plasmid of *C. jejuni* YH002 (Figure 1), an in-depth analysis of the frequency of the RA**A**TTY (the predominant motif) demonstrated the existence of both hypomethylated and hypermethylated areas that were associated with specific genomic features/CDS (Supplementary Tables 1, 2). In this analysis, a value of 1 indicated that a single methylated site of the RAATTY motif in a CDS. Values greater than 40 indicated that CDS could be hypermethylated and those less than 1 indicated the possibility of hypomethylated CDSs (Mou et al., 2015). Based on this, CDS within the chromosome of *C. jejuni* YH002 contained an average of 11.1 RAATTY motifs per CDS (Figure 2B). However, certain CDS contained ≥ 60 RAATTY motifs, with the number of RAATTY motifs highest for a putative type IIS restriction-modification enzyme (78), a possible restriction-modification protein (64), the glutamate synthase [NADPH] large chain (60), and a DNA methylase (66) encoded for the plasmid (Supplementary Tables 1, 2). On the other extreme were the genes encoding tRNA, with a RAATTY frequency of 0.1 (Figure 2B), which indicated that many tRNA genes did not contain this methylation motif (Supplementary Table 1). Both integrated genetic elements (phage/CJIE1 and CJIE) and rRNAs had a considerably lower frequency of RAATTY compared to the chromosome and plasmid.

### Comparative Analysis of Methylation in the Genomes of *C. jejuni* YH002, *C. jejuni* 81–176, and *C. jejuni* NCTC 11168

**FIGURE 3:**
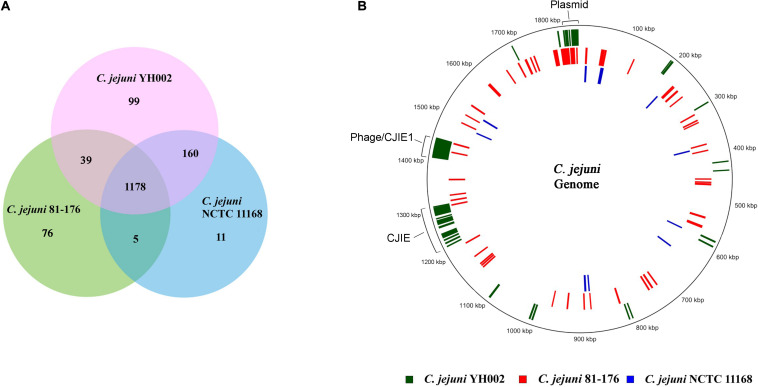
Comparative analysis of the methylome of the *C. jejuni* strains YH002, 81–176 and NCTC 11168. The Venn diagram **(A)** depicts the overlapping and unique CDSs methylated among *C. jejuni* YH002, *C. jejuni* 81–176, and *C. jejuni* NCTC 11,168, while the schematic **(B)** shows the relative locations of the uniquely methylated CDSs in the three *C. jejuni* genomes. The locations of the plasmid-borne genes are also shown.

For the comparative analysis of the methylation patterns, previously reported methylation motifs from the REBASE database for the *C. jejuni* strains 81–176 and NCTC 11168 were obtained and the methylated CDS in all three genomes identified. Analysis of these CDSs revealed that a majority of the methylated CDSs (*n* = 1,178) were common among the three strains, though some uniquely methylated CDSs were also identified in each genome: 99 for *C. jejuni* YH002, 76 for *C. jejuni* 81–176, and 11 for *C. jejuni* NCTC 11168 (Figure 3A and Table 2). Mapping of these uniquely methylated CDSs revealed discernible patterns (Figure 3B) with the uniquely methylated genes more uniformly distributed in the genome of *C. jejuni* 81–176 compared to the other two genomes (YH002 and NCTC 11168).

**TABLE 2:** Proteins encoded by CDS uniquely* methylated amongst the three strains of *C. jejuni* YH002, 81–176, and NCTC 11168.

*C. jejuni* YH002	*C. jejuni* 81–176	*C. jejuni* NCTC11168

Chromosome		
TnpV	Protein of unknown function DUF262 family	ATP synthase protein I
Mobile element protein	Cytochrome c, putative	Conserved hypothetical protein
Peroxide stress regulator; Ferric uptake regulation protein; Fe2 + /Zn2 + uptake regulation proteins	Thiol:disulfide interchange protein (dsbC), putative	Drug resistance transporter, Bcr/CflA family
FIG01371694: hypothetical protein	Gamma-glutamyltranspeptidase	FIG001196: Membrane protein YedZ
tRNA-Thr-TGT	LSU ribosomal protein L21p	FIG00469472: hypothetical protein
FIG00470965: hypothetical protein	FIG00470268: hypothetical protein	FIG00469493: hypothetical protein
Family of unknown function (DUF450) family	Predicted amino-acid acetyltransferase complementing ArgA function in Arginine Biosynthesis pathway	hypothetical protein
First ORF in transposon ISC1904	Small-conductance mechanosensitive channel	L-threonine 3-O-phosphate decarboxylase
ATP synthase protein I-like membrane protein	Putative helix-turn-helix motif protein	Nicotinate-nucleotide adenylyltransferase/Iojap protein
Para-aminobenzoate synthase, amidotransferase component	Peptidyl-tRNA hydrolase	Para-aminobenzoate synthase, aminase component
Hypothetical domain/C-terminal domain of CinA type S	Ubiquinone/menaquinone biosynthesis methyltransferaseUbiE @ 2-heptaprenyl-1,4-naphthoquinone methyltransferase	Putative type IIS restriction/modification enzyme, C-terminal half
Putative Dihydrolipoamide dehydrogenase; Mercuric ion reductase; PF00070 family, FAD-dependent NAD(P)-disulphideoxidoreductase	LSU ribosomal protein L32p	−
FIG00471123: hypothetical protein	6,7-dimethyl-8-ribityllumazine synthase	−
tRNA-Phe-GAA	FIG00469601: hypothetical protein	−
FIG00471396: hypothetical protein	FIG00388607: hypothetical protein	−
tRNA-Ser-TGA	FIG00469384: hypothetical protein	−
**CJIE in chromosome:**	tRNA-Gly-TCC	−
FIG00469806: hypothetical protein	LSU ribosomal protein L33p	−
Death on curing protein, Doc toxin	UbiD family decarboxylase associated with menaquinone via futalosine	−
Thymidine kinase	FIG00470496: hypothetical protein	−
FIG00470782: hypothetical protein	FIG00470712: hypothetical protein	−
Phage antirepressor protein	FIG00471113: hypothetical protein	−
TraD, putative	FIG00472079: hypothetical protein	−
HD domain protein	FIG00469622: hypothetical protein	−
M protein, putative	FIG00470120: hypothetical protein	−
pilT protein, putative	FIG00469655: hypothetical protein	−
Hypothetical phage protein, putative	FIG00470478: hypothetical protein	−
FIG00470684: hypothetical protein	FIG00469686: hypothetical protein	−
IncF plasmid conjugative transfer pilus assembly protein TraF	tRNA-Gly-GCC	−
IncF plasmid conjugative transfer pilus assembly protein TraW	4-methyl-5(beta-hydroxyethyl)-thiazole monophosphate synthesis protein	−
IncF plasmid conjugative transfer pilus assembly protein TraU	FIG00469538: hypothetical protein	−
Transcriptional regulator, XRE family	LSU ribosomal protein L34p	−
IncF plasmid conjugative transfer surface exclusion protein TraT	Beta-1,4-N-acetylgalactosaminyltransferase	−
Thioredoxin, putative	Beta-1,4-galactosyltransferase	−
FIG00471674: hypothetical protein	ATP synthase F0 sector subunit a	−
Domain of unknown function (DUF332) superfamily	N-Acetyl-neuraminatecytidylyl transferase	−
FIG00471014: hypothetical protein	Peptidyl-prolylcis-trans isomerase	−
IncF plasmid conjugative transfer pilus assembly protein TraH	Menaquinone via 6-amino-6-deoxyfutalosine step 1	−
IncF plasmid conjugative transfer pilus assembly protein TraC	Protein containing aminopeptidase domain	−
Phosphonate ABC transporter phosphate-binding periplasmic component (TC 3.A.1.9.1)	Formyltransferase domain protein	−
FIG00470688: hypothetical protein	MGC82361 protein	−
Conjugative transfer protein TraV	tRNA-Ser-GCT	−
Thiol:disulfide involved in conjugative transfer	Flagellar biosynthesis protein FliL	−
IncF plasmid conjugative transfer pilus assembly protein TraB	GDP-mannose 4,6-dehydratase	−
IncF plasmid conjugative transfer pilus assembly protein TraK	LSU ribosomal protein L13p (L13Ae)	−
IncF plasmid conjugative transfer pilus assembly protein TraE	FIG00626672: hypothetical protein	−
IncF plasmid conjugative transfer pilus assembly protein	ATP/GTP-binding protein	−
FIG00471953: hypothetical protein	Anaerobic dimethyl sulfoxide reductase chain A	−
F-box DNA helicase 1	Anaerobic dimethyl sulfoxide reductase chain B	−
**Phage/CJIE1 in chromosome:**	Anaerobic dimethyl sulfoxide reductase chain C	−
FIG00471569: hypothetical protein	Ribonuclease HI	−
FIG00471570: hypothetical protein	RepA protein homolog	−
DNA adenine methylase	tRNA-Leu-TAG	−
Phage virion morphogenesis protein, putative	Mll5128 protein	−
Phage tail length tape-measure protein	FIG00470615: hypothetical protein	−
Phage major tail tube protein, putative	LSU ribosomal protein L4p (L1e)	−
Major tail sheath protein	Lipopolysaccharide core biosynthesis protein LpsA	−
FIG00470503: hypothetical protein	tRNA-Ala-GGC	−
FIG00469719: hypothetical protein	**Plasmid:**	−
FIG00470940: hypothetical protein	FIG00471024: hypothetical protein	−
Tail fiber protein H, putative	DNA transformation competency	−
Baseplate assembly protein J, putative	Type IV secretion/competence protein (VirB9)	−
Baseplate assembly protein W, putative	Type II/IV secretion system ATP hydrolase TadA/VirB11/CpaF, TadA subfamily	−
FIG00470824: hypothetical protein	FIG00469544: hypothetical protein	−
Baseplate assembly protein V	FIG00469919: hypothetical protein	−
FIG00470123: hypothetical protein	Hypothetical protein pVir0008	−
Phosphonate ABC transporter phosphate-binding periplasmic component (TC 3.A.1.9.1)	Hypothetical protein pVir0010	−
FIG00470179: hypothetical protein	Hypothetical protein pVir0012	−
FIG00471901: hypothetical protein	Hypothetical protein pVir0015	−
Mu-like prophage I protein, putative	Hypothetical protein pVir0016	−
FIG00469991: hypothetical protein	RepE replication protein, putative	−
FIG00472447: hypothetical protein	FIG00470162: hypothetical protein	−
Phage protein	FIG00472444: hypothetical protein	−
FIG00471796: hypothetical protein	FIG00472140: hypothetical protein	−
Prophage MuSo1, F protein, putative	Toxin-antitoxin protein, putative	−
Phage tail protein, putative	−	−
Putative regulator of late gene expression	−	−
DNA-binding protein, putative	−	−
Endonuclease I precursor	−	−
FIG00470704: hypothetical protein	−	−
FIG00471749: hypothetical protein	−	−
FIG00469659: hypothetical protein	−	−
Host-nuclease inhibitor protein Gam, putative	−	−
FIG00471516: hypothetical protein	−	−
FIG00470000: hypothetical protein	−	−
DNA transposition protein	−	−
Bacteriophage DNA transposition protein A, putative	−	−
FIG00470129: hypothetical protein	−	−
Phage repressor protein, putative	−	−
FIG00469983: hypothetical protein	−	−
**Plasmid:**	−	
Aminoglycoside phosphotransferase	−	−
FIG00472625: hypothetical protein	−	−
FIG00470991: hypothetical protein	−	−
FIG00469861: hypothetical protein	−	−
IncQ plasmid conjugative transfer protein TraG	−	−
FIG00471323: hypothetical protein	−	−
FIG00470038: hypothetical protein	−	−
FIG00469571: hypothetical protein	−	−
FIG00470457: hypothetical protein	−	−
Cag pathogenicity island protein (cag12)	−	−
FIG00472706: hypothetical protein	−	−

*Unique methylation is defined as CDS methylated in only one of the three genomes.

Both *C. jejuni* YH002 and *C. jejuni* 81–176 have a collection of plasmid-borne genes that are not present in *C. jejuni* NCTC 11168. Interestingly, these plasmid-borne genes are also methylated (Figure 3B). The other two uniquely methylated regions in *C. jejuni* YH002 are both located in the integrated genetic elements (CJIEs). For *C. jejuni* YH002, this finding seems conflicted by the identification of proteins related to various components of putative RM systems, including Type I RM systems (Ghatak et al., 2020) and the presence of the genes encoding McrBC, which is responsible for specific restriction activity against methylated bases (usually 5 mC or 5 hmC).

### Analysis of Methylation Locations in Gene Promoters

To enhance our ability to define genes that may be regulated by methylation, methylated sites located in gene promoter regions were identified by searching the known methylated sites within the −50 nt promoter sequences obtained by sequence mapping to the *C. jejuni* NCTC 11168 promoters (Dugar et al., 2013). Methylated bases were found in the promoter regions of 18 different hypothetical genes and 123 genes with predicted functions (Supplementary Table 3). Interestingly, several virulence genes were found to contain methylated nucleotides within their promoter region. This finding indicates a role for methylation in the transcription regulation of these genes, which includes *flhB*, *rpoN*, *kpsD*, multidrug resistance genes, and CRISPRs.

Further analysis of these promoter regions revealed the existence of all 6 different methylation motifs. Once again, RA**A**TTY was the most dominant motif, having been identified 76 times within the predicted methylated promoters. The remaining motifs were found much less frequently, with CCYG**A** identified twice, GKA**A**YG identified three times, **A**CNNNNNCTC/G**A**GNNNNNGT identified once, TA**A**YNNNNNTGC/GC**A**NNNNNRTTA identified seven time, and the CGCG**A** motif identified twice. The remaining methylated promoter regions did not appear to contain any of these defined methylation motifs, suggesting additional methylation motifs remain to be discovered and/or some of the six motifs extend beyond the −50 nt promoter sequences.

## Discussion

Methylation of the genome plays several important roles in prokaryotic biology including acting as a defense mechanism against the incorporation of intruding nucleic acids into the bacterial host DNA and the regulation of gene expression by influencing transcription, replication initiation and mismatch repair (Murray et al., 2012; Mou et al., 2015; Blow et al., 2016). At the heart of host defense mechanisms utilizing methylation of host DNA is the restriction-modification (RM) system comprised of a restriction endonuclease (REase) and methyltransferase (MTase) (Murray et al., 2012; Blow et al., 2016). Of the four types (I–IV) of RM systems that have been reported, a majority have been classified as Type II systems (Blow et al., 2016). This is believed to be the result of technological difficulties in detecting the other RM systems (type I, type III, type IV) (Murray et al., 2012). However, this bottleneck has been mostly overcome with SMRT sequencing (Rhoads and Au, 2015), which has provided an opportunity to study the methylome of bacteria and contribute to the rapidly growing resource pool of methylation data (Roberts et al., 2015). Nonetheless, to date there are only a few reports on the methylome of *Campylobacter* isolates (Murray et al., 2012; Mou et al., 2015; O’Loughlin et al., 2015; Zautner et al., 2015) and to our knowledge there is no report on the methylome of *C. jejuni* originating from a food source. In our analysis we observed 7 motifs, of which 4 motifs were part of two bipartite pairs. Similar findings have been documented previously (Murray et al., 2012; Mou et al., 2015; O’Loughlin et al., 2015). In addition to the previously reported motifs, our results also identified a novel putative motif CGCG**A** for *Campylobacter* spp. as we did not find a match for this motif within the *Campylobacter* genomes in the REBASE database. However, an exact match with *Streptococcus mutans* was found, possibly indicated shared RM systems even across distant classes of bacteria (Epsilon-proteobacteria and Bacilli). During our analysis, we undertook a comparative modQV analysis to determine the effects of various cut-offs and also to derive a list of motifs with high confidence. Results suggested that comparative mod QV analysis is crucial for motif detection as various modQV cut-off values yielded varying results. Moreover, detection of base modifications by SMRT sequencing is known to produce secondary signals (usually 5 bases upstream m6A; 2 bases upstream 5 mC). Therefore, rigorous validation of software identified motifs is critical for correct motif identification as has been done in the present study.

Visualization of the methylated segments in the *C. jejuni* YH002 genome indicated widespread methylation covering almost 99% of the genome, which has been reported previously (Mou et al., 2015). Comparative analysis of the methylomes of three *C. jejuni* strains (YH002, 81–176 and NCTC 11168) highlighted that methylation patterns within a species are non-uniform and unlike that previously reported by O’Loughlin et al. (2015) we could not convincingly detect the m4C type of modification. However, these results coincide with those of a previous study where only the m6A type modification was documented in *C. jejuni* (Mou et al., 2015).

Detection of the *mcrBC* gene in the *C. jejuni* YH002 genome is also interesting considering this type IV RM system is methylation dependent for its restriction activity, which can restrict the establishment of any foreign DNA from sources such as bacteriophage that lack the proper DNA methylation. It is believed that methylation-specific RM systems such as this have evolved in response to an evolutionary arms race between bacteriophage and bacteria. As bacteriophages evolved mechanisms to allow for the invasion of bacterial cells, host bacteria have armed themselves with methylation-specific RM systems, which then force the bacteriophage to alter their genome if they are to avoid cleavage (Bickle and Kruger, 1993; Labrie et al., 2010; Sukackaite et al., 2012). Therefore, we believe the genome of *C. jejuni* YH002 may be unusual for possessing a type IV RM system (*mcrBC*) while simultaneously harboring two methylated bacteriophage elements. However, taking into account that McrBC only weakly restricts methylcytosines other than hydroxymethylcytosine (Ishikawa et al., 2010) and the suggested absence of m4C type methylation in the present analysis, the co-existence of both the methylated bacteriophage elements and the *mcrBC* system appears justifiable.

Methyltransferases that are typically involved in the regulation of gene expression often do not contain functional restriction-modification enzymes and are therefore referred to as orphan or solitary DNA methyltransferases. Methyltransferases of this type are important in cellular biology because of their ability to activate or silence genes without mutating the gene itself. Orphan methyltransferases can be found in *C. jejuni* YH002. For example, Accession # ARJ53876 (Ghatak et al., 2020) is a 227 amino acid protein found in *C. jejuni* YH002 whose sequence is identical to that of the N^6^-adenine-specific DNA methyltransferase cj1461 (Kim et al., 2008). The presence of orphan methyltransferases and the fact that multiple putative virulence genes were found in this study to contain methylation sites within their promoter regions (Supplementary Table 3) implies the ability of *C. jejuni* YH002 to employ this mode of regulation for the expression of certain virulence genes.

To uncover the genes that contained methylation sites within their promoter regions, the start of transcription was initially assessed. In this manuscript, TSSs were defined using data obtained from previously published RNA-seq experiments performed with biological replicates from specific *C. jejuni* strains during the mid-log phase of growth (Dugar et al., 2013). Although analysis in this fashion allowed the designation of a TSS to be based around experimental evidence instead of simply computational formulations, it is highly possible that some sites are missing from this analysis. For example, additional transcripts that were not assessed here may be produced by *C. jejuni* strains grown under alternative conditions. In addition, because the analysis relies upon those transcripts identified in *C. jejuni* NCTC 11,168 (which shares a high sequence similarity with *C. jejuni* YH002), transcripts unique to *C. jejuni* YH002 (e.g., CJIEs) would not be accounted for using this method. Regardless, TSSs regulated by σ^70^ as well as σ^28^ and σ^54^ were established using this method. It is also important to note that transcripts corresponding to many of the areas designated here were found in other *C. jejuni* strains including *C. jejuni* 81–176, *C. jejuni* 81116, and *C. jejuni* RM1221 using RNA-seq (Dugar et al., 2013), which helps highlight the reliability of the TSS predictions used.

Of the virulence genes identified that contained a methylation site within its promoter region, one appeared to be directly related to flagellum production (*flhB).* Much is known about the biopolar flagella in *C. jejuni.* With approximately 25–30 proteins involved in the assembly and function of flagella (Lertsethtakarn et al., 2011), this system was initially classified as a virulence factor based upon its role in the motility and the invasion of epithelial cells in the intestine (Morooka et al., 1985; Black et al., 1988; Wassenaar et al., 1991; Takata et al., 1992; Nachamkin et al., 1993). Additional investigations have shown these genes are also important not only for the secretion of non-flagellar proteins involved in virulence but that changes to the glycan composition of the flagella can affect autoagglutination, a preliminary step in biofilm formation, and evasion of the host’s immune response (Guerry, 2007). Insertional inactivation of *flhB* in *C. jejuni* resulted in mutants which were non-motile and had an altered cellular morphology (Matz et al., 2002). These mutants showed a significant reduction in the level of *flaA* transcript, demonstrating a role for FlhB in the regulation of *flaA* and thus indicating that the impact methylation has on the transcriptome may extend beyond those genes directly expressed from methylated promoters.

Another virulence gene in *C. jejuni* YH002 with widespread effects whose transcription is putatively regulated by DNA methylation is *rpoN*, which encodes for the σ^54^ protein. In general, sigma factors are global regulators involved with the recruitment of RNA polymerase to the promoter regions in which they are bound. Like other *C. jejuni* strains, *C. jejuni* YH002 appears to encode three sigma factors (Parkhill et al., 2000; Ghatak et al., 2020): (1) *rpoD*, which is considered the “housekeeping” sigma factor; (2) *fliA*, which regulates the flagellar operon;, and (3) *rpoN*, which has long been associated with a range of physiological phenomena and has recently been designated as a “central controller of the bacterial exterior” (Francke et al., 2011). Previous studies in *C. jejuni* have shown that not only is *rpoN* required for the transcription of a number of genes involved in bacterial motility (Hendrixson et al., 2001) but that *rpoN* mutants also lacked the presence of secreted proteins and are attenuated in their ability to both adhere to and be internalized by HeLa cells (Fernando et al., 2007). Interestingly, a transcriptional activator associated with σ^54^ was also found to contain a methylation site within its promoter; further establishing the importance of methylation for proper regulation of σ^54^ in *C. jejuni.*

Identification of a methylation site within the promoter regions of genes encoding the capsular polysaccharide export protein (KpsD), clustered regularly interspaced short palindromic repeats (CRISPR) genes, and a putative multidrug efflux pump are also worth mentioning because of their contributions to the survival, adaptation, and antibiotic resistance of *C. jejuni* (Bacon et al., 2001; Luangtongkum et al., 2009; Guerry et al., 2012; Louwen et al., 2014). Considering the multifaceted roles of these genes in virulence, the information presented here regarding methylation as a possible mode of regulation will be highly important for combating diarrheal disease caused by *C. jejuni* since its ability to successfully infect a hosts lies not only in the presence of specific virulence genes but also in the pathogen’s capacity to appropriately regulate those genes. Overall, studies such as this, which expand upon our knowledge of DNA methylation, serve to increase our understanding of how bacterial genomes are able to adapt to changes in the environment while simultaneously defending the integrity of the genetic material critical for their survival.

## Data Availability Statement

Publicly available datasets were analyzed in this study. This data can be found here: GenBank of NCBI with the accession Nos. CP020776 and CP020775.

## Author Contributions

CA and YH planned and analyzed experiments, authored, and edited the manuscript. SG planned, conducted, analyzed experiments, authored, and edited the manuscript. SR planned, conducted, analyzed experiments, and edited the manuscript. All authors contributed to the article and approved the submitted version.

## Conflict of Interest

The authors declare that the research was conducted in the absence of any commercial or financial relationships that could be construed as a potential conflict of interest.

## Footnotes

1 http://tools.neb.com/genomes/index.php?page=C

2 http://www.pacb.com/support/software-downloads/

3 https://github.com/PacificBiosciences/MotifMaker

4 https://rast.nmpdr.org/

5 https://github.com/PacificBiosciences/Bioinformatics-Training/wiki/

## References

[B1] Bacon D. J. Alm R. A. Hu L. Hickey T. E. Ewing C. P. Batchelor R. A. (2002). DNA sequence and mutational analyses of the pVir plasmid of *Campylobacter jejuni* 81-176. *Infect. Immun.* 70 6242–6250. 10.1128/Iai.70.11.6242-6250.2002 12379703 PMC130303

[B2] Bacon D. J. Szymanski C. M. Burr D. H. Silver R. P. Alm R. A. Guerry P. (2001). A phase-variable capsule is involved in virulence of *Campylobacter jejuni* 81-176. *Mol. Microbiol.* 40 769–777. 10.1046/j.1365-2958.2001.02431.x 11359581

[B3] Bickle T. A. Kruger D. H. (1993). Biology of DNA restriction. *Microbiol. Rev.* 57 434–450. 10.1128/Mmbr.57.2.434-450.1993 8336674 PMC372918

[B4] Black R. E. Levine M. M. Clements M. L. Hughes T. P. Blaser M. J. (1988). Experimental *Campylobacter jejuni* infection in humans. *J. Infect. Dis.* 157 472–479. 3343522 10.1093/infdis/157.3.472

[B5] Blow M. J. Clark T. A. Daum C. G. Deutschbauer A. M. Fomenkov A. Fries R. (2016). The epigenomic landscape of prokaryotes. *PLoS Genet.* 12:e1005854. 10.1371/journal.pgen.1005854 26870957 PMC4752239

[B6] Dugar G. Herbig A. Forstner K. U. Heidrich N. Reinhardt R. Nieselt K. (2013). High-resolution transcriptome maps reveal strain-specific regulatory features of multiple *Campylobacter jejuni* isolates. *PLoS Genet.* 9:e1003495. 10.1371/journal.pgen.1003495 23696746 PMC3656092

[B7] Facciola A. Riso R. Avventuroso E. Visalli G. Delia S. A. Lagana P. (2017). *Campylobacter*: from microbiology to prevention. *J. Prev. Med. Hyg.* 58 E79–E92. 28900347 PMC5584092

[B8] Fernando U. Biswas D. Allan B. Willson P. Potter A. A. (2007). Influence of *Campylobacter jejuni fliA, rpoN* and *flgK* genes on colonization of the chicken gut. *Int. J. Food Microbiol.* 118 194–200. 10.1016/j.ijfoodmicro.2007.07.038 17761334

[B9] Francke C. Kormelink T. G. Hagemeijer Y. Overmars L. Sluijter V. Moezelaar R. (2011). Comparative analyses imply that the enigmatic sigma factor 54 is a central controller of the bacterial exterior. *BMC Genomics* 12:385. 10.1186/1471-2164-12-385 21806785 PMC3162934

[B10] Ghatak S. He Y. P. Reed S. Irwin P. (2020). Comparative genomic analysis of a multidrug-resistant *Campylobacter jejuni* strain YH002 isolated from retail beef liver. *Foodborne Pathog. Dis.* 17 576–584. 10.1089/fpd.2019.2770 32077758

[B11] Grant C. E. Bailey T. L. Noble W. S. (2011). FIMO: scanning for occurrences of a given motif. *Bioinformatics* 27 1017–1018. 10.1093/bioinformatics/btr064 21330290 PMC3065696

[B12] Guerry P. (2007). *Campylobacter* flagella: not just for motility. *Trends Microbiol.* 15 456–461. 10.1016/j.tim.2007.09.006 17920274

[B13] Guerry P. Poly F. Riddle M. Maue A. C. Chen Y. H. Monteiro M. A. (2012). *Campylobacter* polysaccharide capsules: virulence and vaccines. *Front. Cell. Infect. Microbiol.* 2:7. 10.3389/fcimb.2012.00007 22919599 PMC3417588

[B14] He Y. P. Yao X. M. Gunther N. W. Xie Y. P. Tu S. I. Shi X. M. (2010). Simultaneous detection and differentiation of C*ampylobacter jejuni, C. coli*, and *C. lari* in chickens using a multiplex real-time PCR assay. *Food Anal. Methods* 3 321–329. 10.1007/s12161-010-9136-6

[B15] Hendrixson D. R. Akerley B. J. DiRita V. J. (2001). Transposon mutagenesis of *Campylobacter jejuni* identifies a bipartite energy taxis system required for motility. *Mol. Microbiol.* 40 214–224. 10.1046/j.1365-2958.2001.02376.x 11298288

[B16] Ishikawa K. Fukuda E. Kobayashi I. (2010). Conflicts targeting epigenetic systems and their resolution by cell death: novel concepts for methyl-specific and other restriction systems. *DNA Res.* 17 325–342. 10.1093/dnares/dsq027 21059708 PMC2993543

[B17] Jokinen C. C. Koot J. M. Carrillo C. D. Gannon V. P. Jardine C. M. Mutschall S. K. (2012). An enhanced technique combining pre-enrichment and passive filtration increases the isolation efficiency of *Campylobacter jejuni* and *Campylobacter coli* from water and animal fecal samples. *J. Microbiol. Methods* 91 506–513. 10.1016/j.mimet.2012.09.005 22985716

[B18] Kim J. S. Li J. Q. Barnes I. H. A. Baltzegar D. A. Pajaniappan M. Cullen T. W. (2008). Role of the *Campylobacter jejuni* cj1461 DNA methyltransferase in regulating virulence characteristics. *J. Bacteriol.* 190 6524–6529. 10.1128/Jb.00765-08 18689478 PMC2565991

[B19] Krzywinski M. Schein J. Birol I. Connors J. Gascoyne R. Horsman D. (2009). Circos: an information aesthetic for comparative genomics. *Genome Res.* 19 1639–1645. 10.1101/gr.092759.109 19541911 PMC2752132

[B20] Labrie S. J. Samson J. E. Moineau S. (2010). Bacteriophage resistance mechanisms. *Nat. Rev. Microbiol.* 8 317–327. 10.1038/nrmicro2315 20348932

[B21] Lertsethtakarn P. Ottemann K. M. Hendrixson D. R. (2011). Motility and chemotaxis in *Campylobacter* and *Helicobacter*. *Annu. Rev. Microbiol.* 65 389–410. 10.1146/annurev-micro-090110-102908 21939377 PMC6238628

[B22] Llarena A. K. Taboada E. Rossi M. (2017). Whole-genome sequencing in epidemiology of *Campylobacter jejuni* infections. *J. Clin. Microbiol.* 55 1269–1275. 10.1128/Jcm.00017-17 28249998 PMC5405246

[B23] Louwen R. Staals R. H. Endtz H. P. van Baarlen P. van der Oost J. (2014). The role of CRISPR-Cas systems in virulence of pathogenic bacteria. *Microbiol. Mol. Biol. Rev.* 78 74–88. 10.1128/MMBR.00039-13 24600041 PMC3957734

[B24] Luangtongkum T. Jeon B. Han J. Plummer P. Logue C. M. Zhang Q. J. (2009). Antibiotic resistance in *Campylobacter*: emergence, transmission and persistence. *Future Microbiol.* 4 189–200. 10.2217/17460913.4.2.189 19257846 PMC2691575

[B25] Matz C. van Vliet A. H. M. Ketley J. M. Penn C. W. (2002). Mutational and transcriptional analysis of the *Campylobacter jejuni* flagellar biosynthesis gene *flhB*. *Microbiology (Reading)* 148(Pt 6) 1679–1685. 10.1099/00221287-148-6-1679 12055288

[B26] Morooka T. Umeda A. Amako K. (1985). Motility as an intestinal colonization factor for *Campylobacter jejuni*. *J. Gen. Microbiol.* 131 1973–1980. 4056739 10.1099/00221287-131-8-1973

[B27] Mou K. T. Muppirala U. K. Severin A. J. Clark T. A. Boitano M. Plummer P. J. (2015). A comparative analysis of methylome profiles of *Campylobacter jejuni* sheep abortion isolate and gastroenteric strains using PacBio data. *Front. Microbiol.* 5:782. 10.3389/fmicb.2014.00782 25642218 PMC4294202

[B28] Murray I. A. Clark T. A. Morgan R. D. Boitano M. Anton B. P. Luong K. (2012). The methylomes of six bacteria. *Nucleic Acids Res.* 40 11450–11462. 10.1093/nar/gks891 23034806 PMC3526280

[B29] Nachamkin I. Yang X. H. Stern N. J. (1993). Role of *Campylobacter jejuni* flagella as colonization factors for three-day old chicks: analysis with flagellar mutants. *Appl. Environ. Microbiol.* 59 1269–1273. 10.1128/Aem.59.5.1269-1273.1993 8517729 PMC182076

[B30] O’Loughlin J. L. Eucker T. P. Chavez J. D. Samuelson D. R. Neal-McKinney J. Gourley C. R. (2015). Analysis of the *Campylobacter jejuni* genome by SMRT DNA sequencing identifies restriction-modification motifs. *PLoS One* 10:e0118533. 10.1371/journal.pone.0118533 25695747 PMC4335053

[B31] Parkhill J. Wren B. W. Mungall K. Ketley J. M. Churcher C. Basham D. (2000). The genome sequence of the food-borne pathogen *Campylobacter jejuni* reveals hypervariable sequences. *Nature* 403 665–668. 10.1038/35001088 10688204

[B32] Quinlan A. R. Hall I. M. (2010). BEDTools: a flexible suite of utilities for comparing genomic features. *Bioinformatics* 26 841–842. 10.1093/bioinformatics/btq033 20110278 PMC2832824

[B33] Rhoads A. Au K. F. (2015). PacBio sequencing and its applications. *Genomics Proteomics Bioinformatics* 13 278–289. 10.1016/j.gpb.2015.08.002 26542840 PMC4678779

[B34] Roberts R. J. Vincze T. Posfai J. Macelis D. (2015). REBASE-a database for DNA restriction and modification: enzymes, genes and genomes. *Nucleic Acids Res.* 43 D298–D299. 10.1093/nar/gku1046 25378308 PMC4383893

[B35] Sanchez-Romero M. A. Cota I. Casadesus J. (2015). DNA methylation in bacteria: from the methyl group to the methylome. *Curr. Opin. Microbiol.* 25 9–16. 10.1016/j.mib.2015.03.004 25818841

[B36] Speegle L. Miller M. E. Backert S. Oyarzabal O. A. (2009). Use of cellulose filters to isolate *Campylobacter* spp. from naturally contaminated retail broiler meat. *J. Food Prot.* 72 2592–2596. 10.4315/0362-028x-72.12.2592 20003744

[B37] Sukackaite R. Grazulis S. Tamulaitis G. Siksnys V. (2012). The recognition domain of the methyl-specific endonuclease McrBC flips out 5-methylcytosine. *Nucleic Acids Res.* 40 7552–7562. 10.1093/nar/gks332 22570415 PMC3424535

[B38] Takata T. Fujimoto S. Amako K. (1992). Isolation of nonchemotactic mutants of *Campylobacter jejuni* and their colonization of the mouse intestinal tract. *Infect. Immun.* 60 3596–3600. 10.1128/Iai.60.9.3596-3600.1992 1500167 PMC257366

[B39] van der Woude M. Braaten B. Low D. (1996). Epigenetic phase variation of the *pap* operon in *Escherichia coli*. *Trends Microbiol.* 4 5–9. 10.1016/0966-842x(96)81498-3 8824788

[B40] Wassenaar T. M. Bleuminkpluym N. M. C. Vanderzeijst B. A. M. (1991). Inactivation of *Campylobacter jejuni* flagellin genes by homologous recombination demonstrates ahat *flaA* but not *flaB* is required for invasion. *EMBO J.* 10 2055–2061. 10.1002/j.1460-2075.1991.tb07736.x 2065653 PMC452888

[B41] World Health Organization (2020). *Campylobacter* [Online]. Available online at: https://www.who.int/news-room/fact-sheets/detail/campylobacter (accessed July 26, 2020)

[B42] Zautner A. E. Goldschmidt A. M. Thurmer A. Schuldes J. Bader O. Lugert R. (2015). SMRT sequencing of the *Campylobacter coli* BfR-CA-9557 genome sequence reveals unique methylation motifs. *Bmc Genomics* 16:1088. 10.1186/s12864-015-2317-3 26689587 PMC4687069

